# But One Kind of Dental Caries

**Published:** 1862-08

**Authors:** 


### Editorial.

### BUT ONE KIND OF DENTAL CARIES.

Health is a unit, which is about the same as saying health is health. But is disease a unit? Persons have been found to give an affirmative answer to this question. But such persons agree that while there is but *one* right way, there may be *many* wrong ones. A normal tooth is a normal tooth; but it is capable of undergoing a variety of abnormal changes, differing in nature, as well as in degree of intensity. And what of it? Nothing, certainly, except that, in a prominent place in a well known journal, this remarkable statement is found:

"Some authors have labored to divide caries into several different kinds, but there is but one kind—the only difference is in the degree of intensity with which it makes its progress in different individuals, and at different periods of life, varying with the habits and constitution of the patient."

Notwithstanding this sentence, it is probable that the writer believes that, whatever may be predisposing causes, chemical corrosion is the *immediate* cause of dental caries. If he does not, space can not be spared, just now, to argue with him. It will do for the present to assume as true, that which is believed by all scientifically educated dentists. And if it is true that chemical corrosion is the cause of dental caries, then there must be as many varieties of corrosion as there are corrodents ; for chemical action is definite, alike in its nature and its results. If but one substance, capable of corroding the teeth, gets in contact with them, then there can be but one kind of corrosion of the teeth ; if two, there must be two kinds ; and if three, three kinds ; and so on for quantities. And the results of chemical corrosion do not depend on "the degree of intensity with which it makes its progress," but on the nature of the corrodent and the object corroded. If sulphuric acid corrodes a metal, the metal is oxydized, and a sulphate of the oxyd is formed. This is the inevitable result, let the "degree of intensity" be what it may.

That which is true of the corrosion of a metal is true of the corrosion of a tooth. That different agents must produce different results, is simply a chemical truism. If hydrochloric acid corrodes tooth bone, in the mouth or out of it, it decomposes the carbonate of lime, while it is decomposed itself; and the results are carbonic acid, water, and chloride of calcium, the latter one of the most soluble of salts. At the same time, it forms a soluble compound with, or dissolves, the subphosphate, while it scarcely interferes with the composition of the animal matter of the bone. But if sulphuric acid is the corrodent, the carbonate is decomposed, but instead of the highly soluble chloride, as above, the almost insoluble sulphate of lime is formed, the subphosphate is not dissolved, and the oxygen and hydrogen are taken from the animal portion, leaving the carbon and nitrogen to dispose of themselves. In short, there is more *decomposition*, but less *disintegration* than when hydrochloric acid is the corrodent. These illustrations may suffice for the present.

If the doctrine of the sentence quoted above be true, it follows that but one chemical agent is present in the production of dental caries, and it is probable the writer will have as much difficulty in persuading the profession that this is true, as he would have in convincing us all that there is but one kind of metallic corrosion. He would have this advantage in maintaining the two positions, that "but one kind" of reasoning would be necessary. Think of him lecturing chemists and druggists after this manner:

Some authors have labored to divide the preparations of iron into several kinds, but there is but one kind—the only difference is in the degree of intensity with which the corrosive agent makes its progress in different specimens, and at different seasons of the year, varying with the source and texture of the metal.

And though this imaginary lecture was delivered with much earnestness, and great apparent sincerity, and even though every look and action of the speaker showed that he expected to be believed, the druggist's clerk, like an infidel wretch, shook his head, and inwardly resolved that, notwithstanding all this, if a patron asked for persulphate of iron, he would not give him the sesquioxyd, even though it is prepared with less inconvenience.

The sentence quoted above is, in itself, unworthy of so much attention; but it stands out prominently in a very respectable journal, and is from the pen of one who is, no doubt, paid a respectable salary for writing such sentiments. This will apologize for the extended notice here given it. W.

